# Effectiveness of Chest Compression-Synchronized Ventilation in Patients with Cardiac Arrest

**DOI:** 10.3390/jcm14072394

**Published:** 2025-03-31

**Authors:** Young T. Oh, Choung A. Lee, Hang A. Park, Juok Park, Sola Kim, Hye J. Park, Sangsoo Han, Soonjoo Wang, Jong W. Kim

**Affiliations:** 1 Department of Emergency Medicine, College of Medicine, Chung-Ang University, 84 Heukseok-ro, Dongjak-gu, Seoul 06974, Republic of Korea; powerfreeze@hanmail.net; 2 Department of Emergency Medicine, Chung-Ang University Hospital, 102 Heukseok-ro, Dongjak-gu, Seoul 06973, Republic of Korea; 3 Department of Emergency Medicine, Hallym University Dongtan Sacred Heart Hospital, 7, Keunjaebong-gil, Hwaseong-si 18450, Republic of Korea; hangapark@gmail.com (H.A.P.); juok.park@gmail.com (J.P.); solarsolakim@gmail.com (S.K.); ji4051@hanmail.net (H.J.P.); wsjking@naver.com (S.W.); 4 Soonchunhyang University Hospital Bucheon, Bucheon 14584, Republic of Korea; brayden0819@daum.net; 5 Department of Surgery, Hallym University Dongtan Sacred Heart Hospital, 7, Keunjaebong-gil, Hwaseong-si 18450, Republic of Korea; jong1124@hallym.or.kr

**Keywords:** mechanical ventilation, cardiopulmonary resuscitation, cardiac arrest, oxygen, pressure

## Abstract

**Background/Objectives:** The aim of this study was to determine the optimal ventilation mode during cardiopulmonary resuscitation (CPR) by comparing the effects of chest compression-synchronized ventilation (CCSV) and intermittent positive-pressure ventilation (IPPV) on arterial blood gases. **Methods:** This prospective randomized controlled study included patients presenting with out-of-hospital cardiac arrest who were randomly assigned to the CCSV or IPPV groups. Arterial blood gas analysis was performed at the start of CPR and 10 min after initiating mechanical ventilation. Primary outcomes included changes in the arterial oxygen and carbon dioxide pressures. **Results:** Of the 144 patients with out-of-hospital cardiac arrest, 30 were included in the study, with 15 each assigned to the CCSV and IPPV groups. The median arterial oxygen pressure in the CCSV group was 76.1 [22.8; 260.3 interquartile range], compared with 8.8 [−1.6; 113.9 interquartile range] in the IPPV group (*p* = 0.250). The change in carbon dioxide pressure was −10.3 [−18.3; −2.7 interquartile range] in the CCSV group and −11.5 [−39.5; 5.6 interquartile range] in the IPPV group (*p* = 0.935). Wilcoxon signed-rank test results revealed significant differences in arterial oxygen and carbon dioxide pressure levels before and after treatment in the CCSV group (*p* = 0.026 and 0.048, respectively). However, in the IPPV group, changes in arterial partial pressure of oxygen and carbon dioxide before and after treatment were non-significant (*p* = 0.095 and 0.107, respectively). **Conclusions:** Although CCSV significantly improved oxygenation and ventilation in patients undergoing CPR, it cannot be considered superior to IPPV.

## 1. Introduction

Despite remarkable advances in medicine over the past decades, the survival rate of patients with cardiac arrest remains very low, at 7.8% as of 2022, with only 5.1% achieving favorable neurological outcomes (Cerebral Performance Category 1–2) upon recovery [1]. To improve the outcomes of cardiopulmonary resuscitation (CPR), along with the importance of the chain of survival—such as early recognition of cardiac arrest, high-quality chest compressions, and early defibrillation—adequate ventilation is considered an essential component [2].

According to the current CPR guidelines, when an advanced airway is not in place, the recommended ratio is to perform 30 chest compressions followed by two rescue breaths (30:2). If an advanced airway is inserted, ventilation should be provided at a rate of approximately 10 breaths per minute (one breath every 6 s) while avoiding hyperventilation [3].

Oxygenation is critical during CPR because maximizing oxygen delivery in a state of inadequate circulation is essential for maintaining aerobic metabolism and adenosine triphosphate. High arterial oxygen tension (partial pressure of arterial oxygen [PaO_2_]) does not lead to intracellular or tissue hyperoxemia during cardiac arrest. Therefore, 100% oxygen is recommended to treat cardiac arrest [4]. Conventional synchronous ventilation involves pausing chest compressions to deliver breaths and is typically applied during bag-mask ventilation or mouth-to-mouth resuscitation [3]. While pausing for ventilation may offer advantages in terms of oxygen delivery and reduced intrathoracic pressure, it carries the risk of decreased blood flow due to interruptions in chest compressions. In contrast, asynchronous ventilation allows for the maintenance of a high chest compression fraction and continuous circulation; however, it may lead to increased intrathoracic pressure, potential hemodynamic compromise, and a higher risk of hyperventilation [5].

Owing to concerns regarding the spread of respiratory infections during the COVID-19 pandemic, the International Liaison Committee on Resuscitation recommended the use of personal protective equipment to prevent infection transmission before performing aerosol-generating procedures [6]. Conversely, the use of personal protective equipment may increase rescuer fatigue and, subsequently, reduce the quality of chest compressions. Therefore, it is imperative to consider ventilation strategies [7]. The American Heart Association (AHA) recommends using ventilation methods that minimize the exposure of healthcare workers to aerosols. This includes the utilization of high-efficiency particulate air filters, rapid intubation, and transitioning to mechanical ventilation [8].

**Figure 1:**
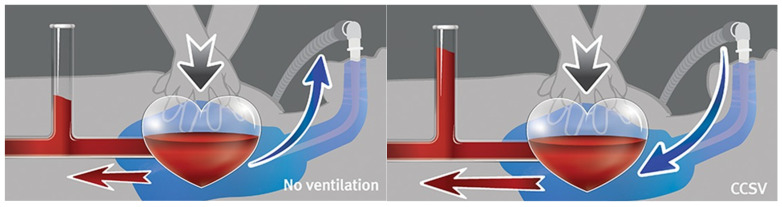
Principle of chest compression synchronized ventilation (CCSV). During chest compressions without ventilatory assistance, the heart is compressed, pushing blood through the aorta (red arrow), while the lungs are also compressed, causing air to be expelled through the airway (blue arrow). In the CCSV mode, ventilatory support is synchronized with chest compressions, increasing intrathoracic pressure at the moment of compression. This synchronization enhances blood ejection, thereby improving circulatory effectiveness.

Chest compression-synchronized ventilation (CCSV) has been proposed as a novel ventilation strategy that minimizes interruptions in chest compressions, prevents excessive increases in intrathoracic pressure caused by the opposing forces of compression and ventilation, and reduces the risk of rescuer infection by maintaining a closed airway system [9]. CCSV delivers synchronized mechanical breaths by inflating the chest during compression, thereby preventing gas from escaping from the thorax and increasing the intrathoracic pressure. This enhances cardiac output through the interaction between the cardiac and thoracic pumps during compression and improves oxygenation owing to increased pressure during simultaneous chest compressions and inspiration. During diastole, no inspiration occurs, preventing the adverse effects of reduced venous return and decreased cardiac output, a concern for hyperventilation (Figure 1) [9].

Comparisons of oxygenation between CCSV and IPPV have only been reported in animal studies. Through this study, we aimed to evaluate whether CCSV can improve oxygenation not only in animals but also in humans.

## 2. Materials and Methods

### 2.1. Study Design and Setting

This was a prospective randomized controlled trial that included patients presenting with out-of-hospital cardiac arrest (OHCA) at a university hospital in Gyeonggi Province. The hospital accommodates approximately 60,000 emergency patients annually, including 320 patients with cardiac arrest. This study was approved by the Institutional Review Board of Hallym University Dongtan Sacred Heart Hospital (HDT 2022-11-011). Informed consent was obtained from the guardians of all patients prior to their inclusion in the study. Furthermore, the study was registered with the Clinical Research Information Service under the registration number KCT0008512. Although study registration was completed after patient enrollment had begun, the registration record accurately reflects the first patient enrollment date. The delay was due to administrative processing, and all research procedures were conducted in full compliance with ethical guidelines.

### 2.2. Study Population

This study included patients aged ≥ 18 years who continued to receive CPR for OHCA and were admitted to the emergency department between February 2023 and June 2024. Patients were excluded from the study if early intubation failed, the patient’s body size was markedly small or large for applying a mechanical compression device, or if arterial blood sampling failed. Patients were also excluded if resuscitation was terminated or return of spontaneous circulation (ROSC) occurred within 10 min after hospital arrival, leading to the cessation of CPR.

### 2.3. Study Protocol

**Figure 2:**
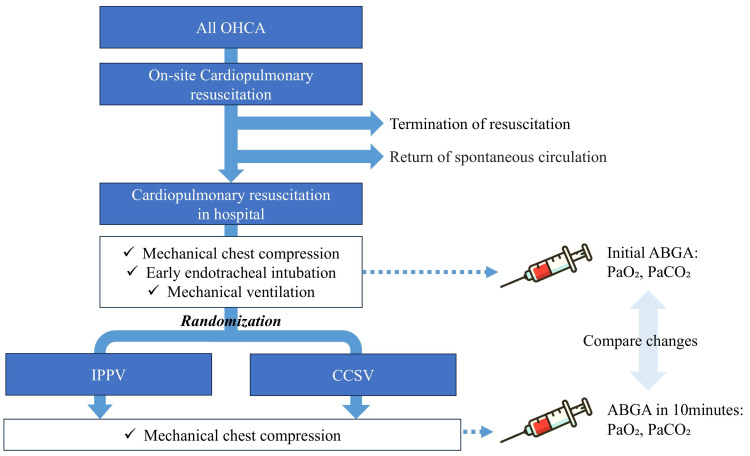
Study protocol. ABGA, arterial blood gas analysis; CCSV, chest compression-synchronized ventilation; IPPV, intermittent positive-pressure ventilation; PaO_2_, partial pressure of arterial oxygen; PaCO_2_, partial pressure of arterial carbon dioxide.

Upon arrival of the study participants, early endotracheal intubation and application of mechanical chest compression devices (LUCAS™ 3, Redmond, WA, USA) were performed to standardize the chest compression rate and depth and to minimize interruptions. This was followed by random allocation to one of the two mechanical ventilation methods (Medumat Transport™, Weinmann GmbH, Hamburg, Germany). Permuted block randomization with blocks of 2 was used for assignment. Arterial blood gas analysis (ABGA) was conducted at the time of intubation (baseline) and repeated at 10 min post-application. Furthermore, arterial blood samples were drawn from the radial or femoral arteries by emergency room physicians (Figure 2).

In the intermittent positive-pressure ventilation (IPPV) group, the settings were as follows: fraction of inspired oxygen, 1; tidal volume, 500 mL; respiratory rate, 10 breaths/min; and peak inspiratory pressure, 60 cmH_2_O, with the trigger turned off [5]. In the CCSV group, the settings were as follows: peak inspiratory pressure, 60 cmH_2_O; inspiration time, 205 ms; and fraction of inspired oxygen, 1 [10].

### 2.4. Sample Size

Since no prior human data were available, we estimated the effect size based on previous animal studies [9]. However, recognizing the inherent differences between animal and human studies, we applied a conservative adjustment to reduce the estimated effect size. This ensures that our sample size calculation accounts for the greater variability expected in human participants. To verify the mean differences between the independent groups, the required total sample size was estimated to be 42 cases, considering an effect size of 0.9, a significance level of 0.05, and a power of 0.8. We aimed to collect data from 110 cases, accounting for an anticipated dropout rate of approximately 60%.

### 2.5. Variables

Baseline information was collected for each patient, covering various aspects of patient demographics and clinical care. Patient data included an identification number, date, age, sex, and relevant medical history. Information regarding the cardiac arrest scene included whether the arrest was witnessed, the time of arrest or discovery, the presence of bystander CPR, and any bystander-provided ventilation.

Details of prehospital care were also recorded, including the time of arrival of emergency services (119), time of initial patient contact, patient’s initial rhythm, type of airway management used, time of advanced airway placement, and time of departure from the scene. In-hospital care information was collected, such as the time of hospital arrival, time of endotracheal intubation, time of LUCAS device application for mechanical chest compressions, type of ventilator used, and timing of arterial blood gas analyses (ABGA #1 and ABGA #2).

Presumed causes of cardiac arrest were documented. The primary outcomes measured were changes in PaO_2_ and partial pressure of carbon dioxide (PaCO_2_), recorded immediately upon hospital arrival and 10 min after initiating mechanical ventilation.

### 2.6. Statistical Analysis

Data analysis was performed using R software, version 4.3.3 (R Development Core Team, 2011; R: A language and environment for statistical computing; R Foundation for Statistical Computing, Vienna, Austria. ISBN 3-900051-07-0). Nominal variables were compared using the chi-squared test, and non-parametric continuous variables were compared using the Mann–Whitney U test. Differences between the groups were evaluated to identify significant differences. Additionally, changes in PaO_2_ and PaCO_2_ before and after treatment in the CCSV and IPPV groups were assessed using the Wilcoxon signed-rank test.

## 3. Results

**Figure 3:**
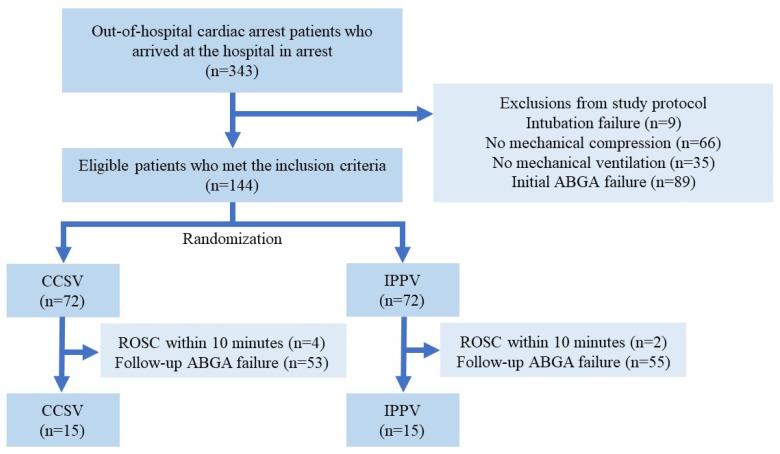
Study flow diagram. ABGA, arterial blood gas analysis; CCSV, chest compression-synchronized ventilation; IPPV, intermittent positive-pressure ventilation; ROSC, return of spontaneous circulation.

During the study period, 343 patients with OHCA were admitted. Of these, 199 were excluded because they did not meet the study protocol criteria, and the remaining 144 were randomized. Among the randomized patients, those who did not have ABGA results or achieved ROSC within 10 min were excluded, resulting in the final inclusion of 15 patients in the CCSV and IPPV groups, respectively (Figure 3).

The general characteristics of the participants are listed in Table 1. The median age was 70 and 63 years, respectively, in the CCSV and IPPV groups, and the proportions of males were 80.0% and 66.7% in the CCSV and IPPV groups, respectively. Furthermore, there were no significant differences in general characteristics between the groups (Table 1).

**Figure 4:**
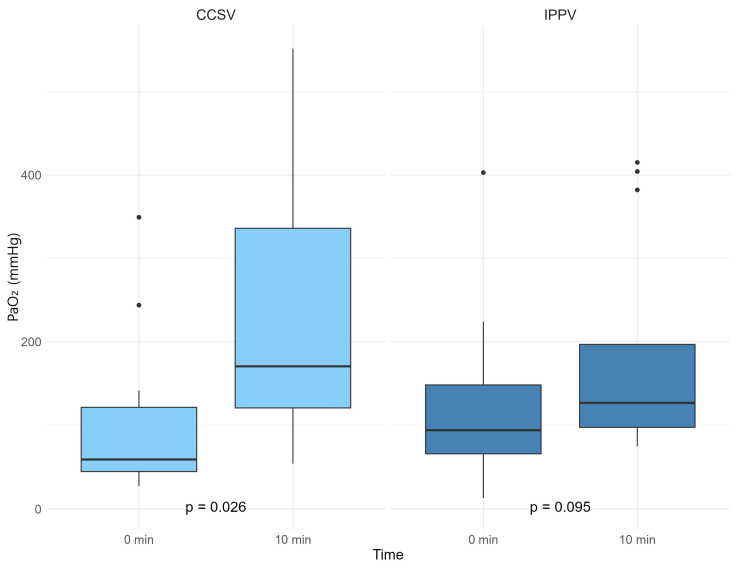
Partial pressure of arterial oxygen (PaO_2_) according to the ventilation method. ABGA, arterial blood gas analysis; CCSV, chest compression-synchronized ventilation; IPPV, intermittent positive-pressure ventilation; PaO_2_, partial pressure of arterial oxygen.

**Figure 5:**
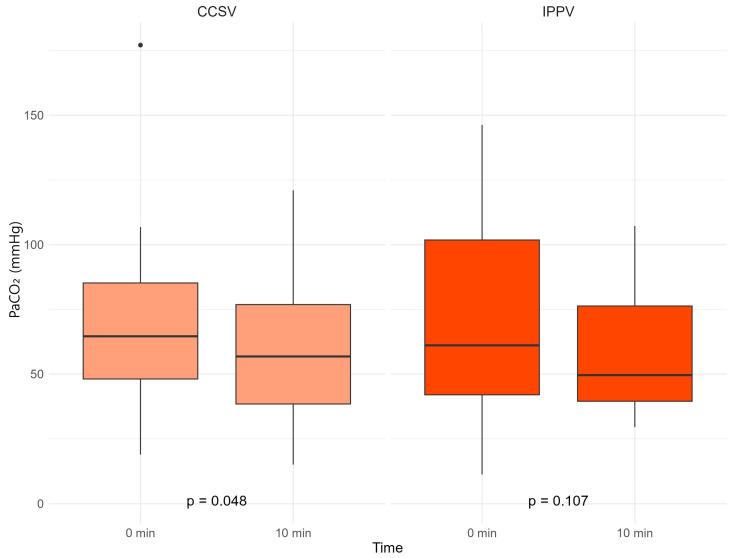
Partial pressure of arterial carbon dioxide (PaCO_2_) according to the ventilation method. ABGA, arterial blood gas analysis; CCSV, chest compression-synchronized ventilation; IPPV, intermittent positive-pressure ventilation; PaCO_2_, partial pressure of arterial carbon dioxide.

The difference in PaO_2_ was 76.1 [22.8; 260.3 interquartile range (IQR)] in the CCSV group and 8.8 [−1.6; 113.9 IQR] in the IPPV group (*p* = 0.250). For PaCO_2_, the difference was −10.3 [−18.3; −2.7 IQR] in the CCSV group and −11.5 [−39.5; 5.6 IQR] in the IPPV group, with a *p*-value of 0.935 (Table 2, Figure 4 and Figure 5).

In the CCSV group, there was a significant difference in PaO_2_ before and after treatment, as determined using the Wilcoxon signed-rank test (*p* = 0.026). The PaCO_2_ also showed a significant difference (*p* = 0.048). In the IPPV group, the change in PaO_2_ before and after treatment was not significant (*p* = 0.095); however, the change in PaCO_2_ was significant (*p* = 0.107) (Figure 4 and Figure 5).

## 4. Discussion

In this study, comparing the ABGA at 0 and 10 min revealed a significant improvement in oxygenation with CCSV, although no such improvement was observed with IPPV.

Recent research and guidelines on cardiac arrest management have focused on the quality of chest compressions, including parameters such as speed, depth, complete chest recoil, and minimization of interruptions as measured using the compression ratio. One study reported that in cases of witnessed cardiac arrest, chest compressions performed by a bystander without rescue breathing resulted in better survival outcomes compared to conventional CPR that included rescue breathing [11]. This is based on the assumption that, in the early phase of cardiac arrest, the lungs and blood contain sufficient residual oxygen to temporarily sustain vital organs. However, as the duration of CPR increases, the importance of ventilation becomes greater. When the CPR duration is extended, ventilated groups reportedly exhibit higher rates of ROSC, survival to discharge, and neurologically intact survival [12].

Evidence regarding the optimal ventilation method for CPR remains inconclusive. In patients with OHCA, continuous ventilation at a rate of 10 breaths per minute without interrupting chest compressions resulted in better survival and neurological recovery rates than the 30:2 compression-to-ventilation ratio group [13]. Conversely, a systematic review in 2017 that analyzed one human study and 10 animal studies found no significant evidence that ventilation at 10 breaths per minute was superior to other ventilation rates [14]. Nevertheless, it is clear that the ideal ventilation strategy should deliver sufficient oxygenation and ventilation while minimizing any interference with the circulation produced by chest compressions.

Passive ventilation has also been proposed as a method to provide oxygenation while maintaining continuous chest compressions. It is based on the principle that during chest compressions, the lungs collapse, and the negative pressure generated by chest recoil causes the lungs to expand, resulting in passive ventilation and gas movement within the airway. However, it has been shown that the amount of ventilation generated by chest compressions alone is insufficient. In a study by Safar et al., chest compression without positive-pressure ventilation produced an average tidal volume of 156 mL in intubated adult patients [15]. In adult patients with OHCA who received compression-only CPR with mechanical chest compression devices, the tidal volume was typically < 20 mL [16,17].

Mechanical ventilation allows for control of the pressure or volume of oxygen delivered during cardiopulmonary resuscitation, enabling the achievement of appropriate tidal volumes. Hernández-Tejedor et al. reported that the use of a mechanical ventilator resulted in better ventilation compared to the use of a bag-valve-mask [18]. Therefore, a synchronized ventilation strategy using a mechanical ventilator may be worth exploring as a novel approach to ventilation during cardiopulmonary resuscitation. Moreover, in the context of infection control raised since the COVID-19 pandemic, it also facilitates the maintenance of a closed system that reduces aerosol generation [8].

In this study, both the CCSV and IPPV groups showed improvements in oxygenation. Unlike a previous study [18] that demonstrated statistically significant improvement in oxygenation with the IPPV mode, our results did not reach statistical significance in that group. However, the CCSV mode resulted in improved oxygenation, which was consistent with the findings of Kill et al. in a porcine model [9]. The difference in statistical significance is likely attributed to the small sample size [19]. Unlike animal studies, research on cardiac arrest in humans inherently involves numerous confounding factors—such as patient age, sex, cause of arrest, medical history, and prehospital variables—which are difficult to control. The effect of CCSV on reducing PaCO_2_ was consistent with findings from animal studies. However, when compared to the IPPV mode, no statistically significant difference was observed, which aligns with the results reported in another study by Kill [20].

The limitations of this study were as follows. First, this study analyzed a limited number of cases in a single-center setting. While our sample size estimation was based on the dropout rates of a pilot study, the actual dropout rate was significantly higher due to the challenges of obtaining arterial blood gas samples at precise time points during ongoing CPR. Second, our study focused on short-term physiological outcomes, such as PaO_2_ and PaCO_2_. Although we recognize the importance of long-term clinical outcomes, including ROSC, survival, and neurological recovery, we were unable to conduct multivariable analyses or assess these broader outcomes due to the inherent challenges of OHCA research and the limited dataset. Third, variability in prehospital care remains an important factor that we were unable to control for in this study. Nevertheless, mechanical chest compression devices were used in both groups to partially control the CPR quality and responder fatigue, ensuring consistency in the compression depth and rate while minimizing interruptions. Fourth, our study did not include a detailed analysis of ventilation parameters, such as tidal volume, inspiratory pressure fluctuations, or minute ventilation, due to technological limitations of the current mechanical ventilators used for CCSV. Lastly, while simple comparative methods were used to analyze changes over time, we acknowledge that statistical approaches such as repeated measures ANOVA or mixed-effects models could provide more robust insights, particularly for evaluating within-subject changes. However, due to the small sample size and the incompleteness of paired data, these methods were not applicable in the current study.

Despite these limitations, our findings highlight the potential of CCSV as a mechanical ventilation strategy during CPR. Accumulating evidence from prospective observational studies suggests that both baseline and dynamic elevations in arterial oxygen content are positively associated with the likelihood of ROSC in a dose-dependent manner [21,22]. Moreover, higher oxygen-carrying capacity has emerged as an independent predictor of favorable neurological outcomes [23]. This study serves as an essential foundation for future research that can expand on these findings with larger sample sizes and more comprehensive clinical endpoints.

## 5. Conclusions

This study demonstrates that CCSV may improve oxygenation and ventilation during CPR compared with IPPV. While these findings indicate that CCSV could be a promising ventilation strategy, the study’s limitations—including the small sample size and potential variability in prehospital factors—warrant further investigation in larger human cohorts. Future research should assess whether CCSV can improve cerebral oxygenation and contribute to better clinical outcomes, such as survival and neurological recovery. By expanding the current understanding of CPR ventilation strategies, this study highlights the need for further research to refine and optimize CPR protocols.

## Figures and Tables

**Table 1:** Baseline characteristics of the study population.

MV Type		CCSV (N = 15)	IPPV (N = 15)	*p*
Age (years)		70.0 [60.5; 80.5]	63.0 [56.5; 79.5]	0.901
Sex	Male	12 (80.0%)	10 (66.7%)	0.680
	Female	3 (20.0%)	5 (33.3%)	
Medical history	HTN	9 (60.0%)	7 (46.7%)	0.714
	DM	6 (40.0%)	6 (40.0%)	1.000
	Heart disease	3 (20.0%)	2 (13.3%)	1.000
	CRF	0 (0.0%)	1 (6.7%)	1.000
	Liver	2 (13.3%)	1 (6.7%)	1.000
Witnessed arrest	Yes	9 (60.0%)	11 (73.3%)	0.699
Bystander CPR	Yes	8 (53.3%)	8 (53.3%)	0.809
	Unknown	2 (13.3%)	1 (6.7%)	
Initial rhythm	VF	1 (6.7%)	2 (13.3%)	0.395
	pVT	0 (0.0%)	0 (0.0%)	
	PEA	7 (46.7%)	9 (60.0%)	
	Asystole	0 (0.0%)	1 (6.7%)	
	Unknown	2 (13.3%)	0 (0.0%)	
Prehospital defibrillation	Yes	2 (13.3%)	3 (20.0%)	1.000
Prehospital airway	BVM	1 (6.7%)	2 (13.3%)	0.659
	Igel	13 (86.7%)	11 (73.3%)	
	Unknown	1 (6.7%)	2 (13.3%)	
Prehospital no-flow time		7.0 [2.0; 10.0]	9.0 [5.0; 11.0]	0.466
Prehospital low-flow time		20.5 [16.5; 27.0]	25.0 [21.0; 31.0]	0.210
Cause of death	Cardiac	6 (40.0%)	6 (40.0%)	0.361
	Trauma	2 (13.3%)	0 (0.0%)	
	Metabolic	3 (20.0%)	4 (26.7%)	
	Respiratory	1 (6.7%)	3 (20.0%)	
	Other	1 (6.7%)	2 (13.3%)	
	Unknown	2 (13.3%)	0 (0.0%)	
Any ROSC	Yes	7 (46.7%)	13 (86.7%)	0.053
ED outcome	Death	9 (60.0%)	7 (46.7%)	0.515
	Admission	6 (40.0%)	7 (46.7%)	
	Transfer	0 (0.0%)	1 (6.7%)	
TTM	Yes	0 (0.0%)	2 (13.3%)	0.464
ECMO	Yes	0 (0.0%)	2 (13.3%)	0.464
Hospital outcome	Death	14 (93.3%)	12 (80.0%)	0.475
	Admission	0 (0.0%)	0 (0.0%)	
	Transfer	1 (6.7%)	2 (13.3%)	
	Discharge	0 (0.0%)	1 (6.7%)	

Data are presented as median (interquartile range) or number (%). MV, mechanical ventilation; CCSV, chest compression-synchronized ventilation; IPPV, intermittent positive-pressure ventilation; HTN, hypertension; DM, diabetes mellitus; CRF, chronic renal failure; VF, ventricular fibrillation; pVT, pulseless ventricular tachycardia; PEA, pulseless electrical activity; BVM, bag-valve-mask; ROSC, return of spontaneous circulation; ED, emergency department; TTM, targeted temperature management; ECMO, extracorporeal membrane oxygenation.

**Table 2:** Arterial blood gas analysis results according to the ventilation method.

MV Type		CCSV (N = 15)	IPPV (N = 15)	*p*
ABGA at 0 min	pH	7.0 [6.9; 7.1]	6.9 [6.9; 7.1]	0.494
	PCO_2_	64.6 [48.1; 85.2]	61.1 [42.0; 101.8]	1.000
	PO_2_	59.0 [43.8; 131.3]	105.9 [73.6; 184.8]	0.267
	HCO_3_	16.4 [13.8; 19.4]	15.5 [12.2; 17.6]	0.678
ABGA at 10 min	pH	7.1 [6.9; 7.2]	7.1 [6.9; 7.2]	0.820
	PCO_2_	56.8 [38.5; 76.8]	49.6 [39.5; 76.3]	0.967
	PO_2_	166.6 [97.7; 303.6]	127.9 [97.8; 335.7]	0.967
	HCO_3_	14.3 [10.4; 16.2]	14.5 [12.4; 18.7]	0.507
ABGA difference	ΔO_2_	76.1 [22.8; 260.3]	8.8 [−1.6; 113.9]	0.250
	ΔCO_2_	−10.3 [−18.3; −2.7]	−11.5 [−36.5; 5.6]	0.935

Data are presented as median (interquartile range). ABGA, arterial blood gas analysis; CCSV, chest compression-synchronized ventilation; IPPV, intermittent positive-pressure ventilation; MV, mechanical ventilation.

## Data Availability

The raw data supporting the conclusions of this article will be made available by the authors on request.

## Abbreviations

The following abbreviations are used in this manuscript:

CPR	cardiopulmonary resuscitation
CCSV	chest compression-synchronized ventilation
IPPV	intermittent positive-pressure ventilation
OHCA	out-of-hospital cardiac arrest
ROSC	return of spontaneous circulation
ABGA	arterial blood gas analysis
